# Successful Distal Radial Artery Puncture for Invasive Arterial Blood Pressure Monitoring in Hemodynamically Unstable Patients

**DOI:** 10.1016/j.jaccas.2025.103317

**Published:** 2025-03-12

**Authors:** Minghao Liu, Hongmei Liu, Yuanyuan Zhao, Hao Zhang, Lei Wang, Feifei Zhang, Jun Zhang, Huanhuan Wang, Xiongwei He, Lijian Gao

**Affiliations:** a Coronary Heart Disease Center, Department of Cardiology, Fuwai Hospital, CAMS & PUMC, Beijing, China; b Department of Nursing, Shihezi People’s Hospital, Shihezi, Xinjiang Uygur Autonomous Region, China; c Department of Cardiology, Shihezi People’s Hospital, Shihezi, Xinjiang Uygur Autonomous Region, China; d Coronary Care Unit, Department of Cardiology, Fuwai Hospital, Chinese Academy of Medical Sciences, Beijing, China; e Shihezi People’s Hospital, Shihezi, Xinjiang Uygur Autonomous Region, China

**Keywords:** acute coronary syndrome, hemodynamics, vascular disease

## Abstract

Invasive arterial blood pressure (ABP) monitoring is essential for critically ill patients. This case series evaluates the efficacy of distal radial artery (dRA) puncture for invasive ABP monitoring, focusing on procedural success, patient outcomes, and procedural limitations. Three patients with acute coronary syndromes underwent dRA puncture for invasive ABP monitoring after percutaneous coronary intervention. The procedures were successful, with no significant complications. The use of dRA preserved proximal radial artery function and improved patient comfort. dRA is a viable alternative to conventional radial artery access for ABP monitoring; the potential benefits include reduced complications and preserved arterial function. The key points to avoid puncture complications include accurately assessing the location of the arterial pulse, using a modified Seldinger technique, avoiding forceful maneuvers, and applying adequate pressure after catheter removal.

## Graphical Abstract

**Figure:**
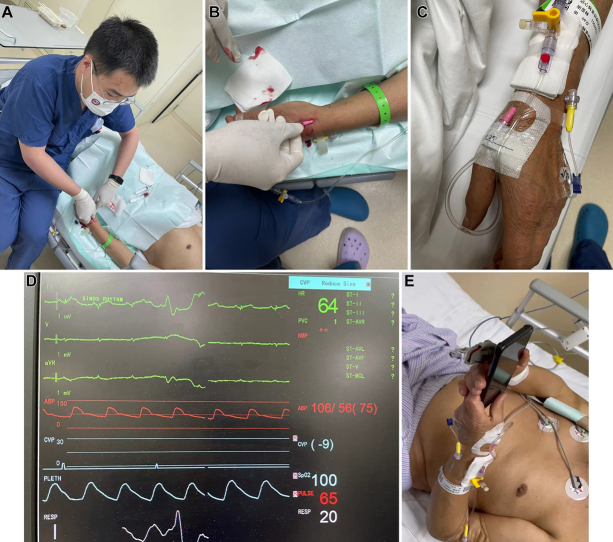

## History of Presentation

### Patient 1

A 67-year-old man with acute anterior wall myocardial infarction underwent emergency revascularization. After percutaneous coronary intervention, the patient required continuous vasopressor support and invasive ABP monitoring because of persistent hypotension.

**Take-Home Messages**

• Distal radial artery puncture offers a reliable alternative to conventional radial artery access for invasive blood pressure monitoring, with fewer complications and preserved arterial function.
• The key points to avoid puncture complications include accurately assessing the location of the arterial pulse, using a modified Seldinger technique, avoiding forceful maneuvers, and applying adequate pressure after catheter removal.

### Patient 2

A 75-year-old man presented with unstable angina. During coronary intervention, attempts to open a chronic total occlusion were unsuccessful. The patient returned to the coronary care unit with an intra-aortic balloon pump in place, requiring invasive ABP monitoring.

### Patient 3

A 66-year-old woman with acute inferior wall myocardial infarction required further invasive ABP monitoring.

## Past Medical Histoy

Patient 1 had a history of coronary artery disease, hypertension, and chronic kidney disease. Patient 2 had a history of hypertension and type 2 diabetes mellitus. Patient 3 had a history of hyperlipidemia and smoking.

## Differential Diagnosis

For all patients, considerations included cardiogenic shock, persistent myocardial ischemia after percutaneous coronary intervention, and hemodynamic instability resulting from acute coronary syndrome.

## Investigations

Continuous invasive blood pressure monitoring was deemed necessary for all patients to manage hypotension and guide therapy, particularly in the context of vasopressor support and mechanical circulatory assistance.

## Management

### Patient 1

**Figure 1:**
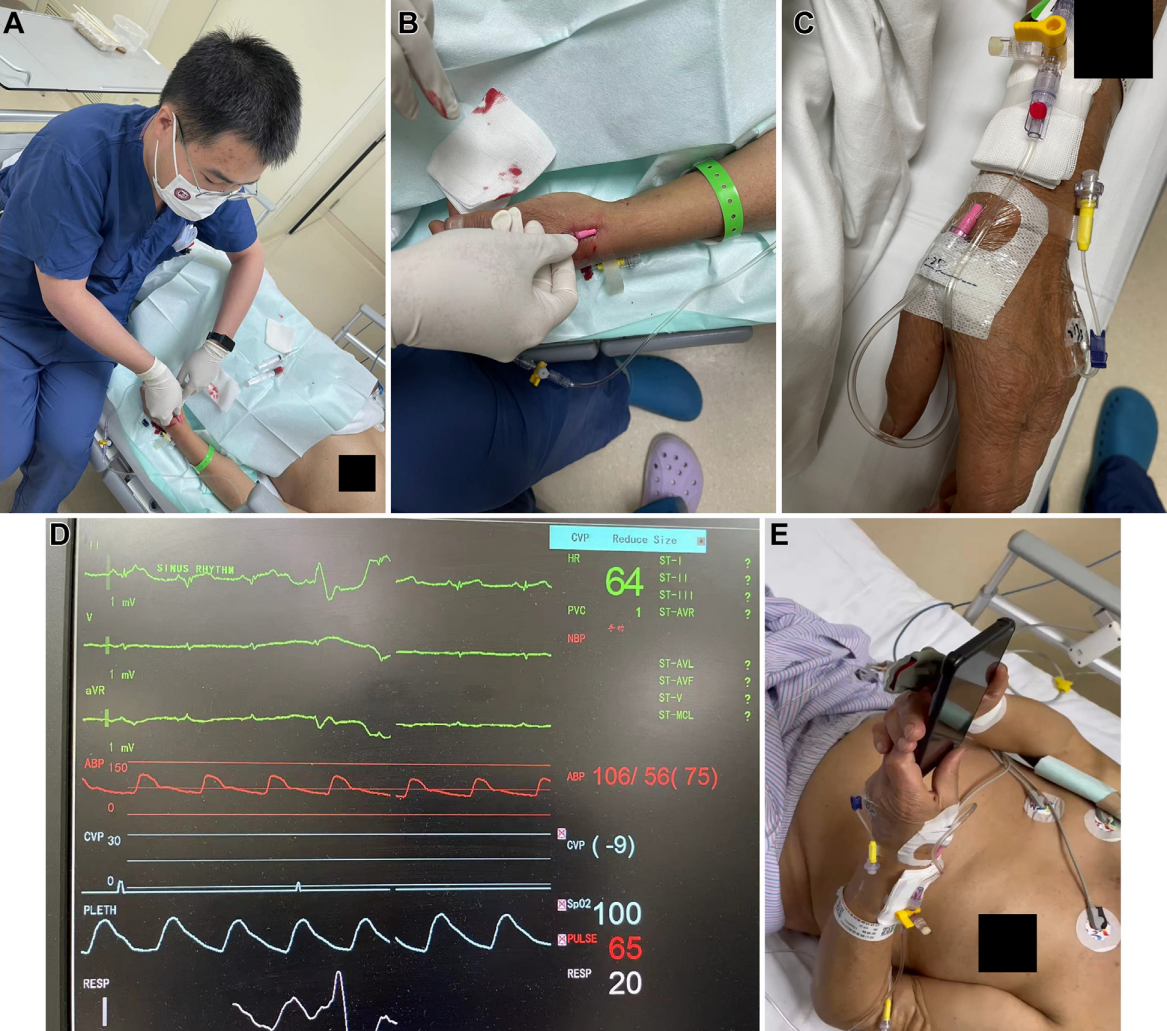
Distal Radial Artery Cannulation for Arterial Pressure Monitoring (Patient 1) (A) Physician performing distal radial artery puncture on patient’s left hand. (B, C) Successful cannulation with arterial catheter connected to pressure transducer. (D) Postprocedural monitoring displaying patient’s vital signs and invasive arterial pressure waveform. (E) After procedure, patient comfortably operates a mobile phone with left hand, demonstrating that the wrist does not require sustained dorsiflexion.

Left dRA puncture was performed with a long catheter, ensuring stable arterial pressure monitoring without unplanned removal or complications. The catheter remained in situ for 3 days (Figure 1).

### Patient 2

**Figure 2:**
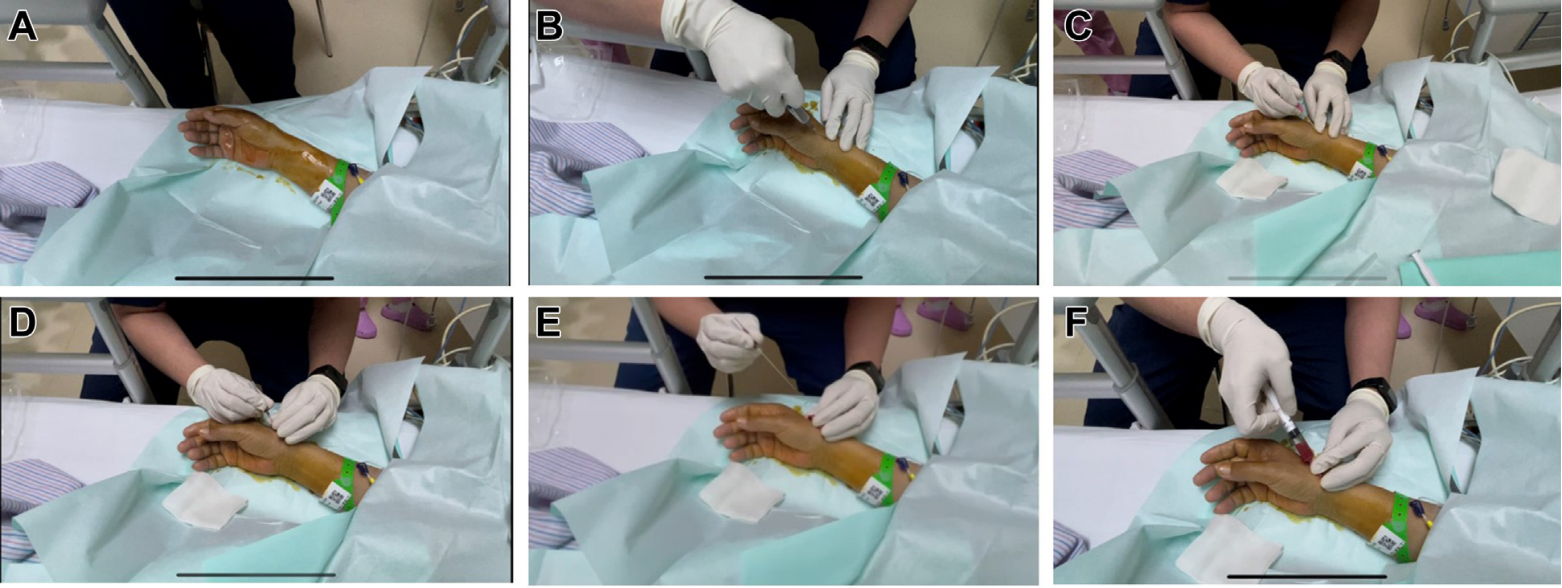
Step-by-Step Demonstration of Distal Radial Artery Cannulation for Invasive Arterial Pressure Monitoring (Patient 2) (A) Puncture site and surrounding area are disinfected with povidone-iodine. (B) After donning sterile gloves, physician administers 1% lidocaine for local anesthesia at puncture site, targeting both superficial and deep tissues. (C) A 20-G arterial catheter is inserted at the point of strongest arterial pulsation. (D) Upon observed blood return, needle core is stabilized, and catheter is advanced. (E) Needle core is removed, and arterial blood flow is confirmed. (F) Catheter is flushed with saline solution using a syringe.

Right dRA puncture was performed using the following steps. 1) For puncture site selection, the strongest pulse point in the anatomical snuffbox was identified. 2) As local anesthesia 2 mL 1% lidocaine was injected subcutaneously at the puncture site. 3) For the puncture technique, a 20-G arterial catheter (BD Angiocath, 1.1 × 48 mm) was inserted at a 30° angle. After blood flow was detected, the catheter was advanced and secured. 4) Catheter maintenance was accomplished by flushing the catheter with saline solution, connecting it to a pressure transducer, and securing it with sterile dressings (Figure 2, Video 1).

### Patient 3

**Figure 3:**
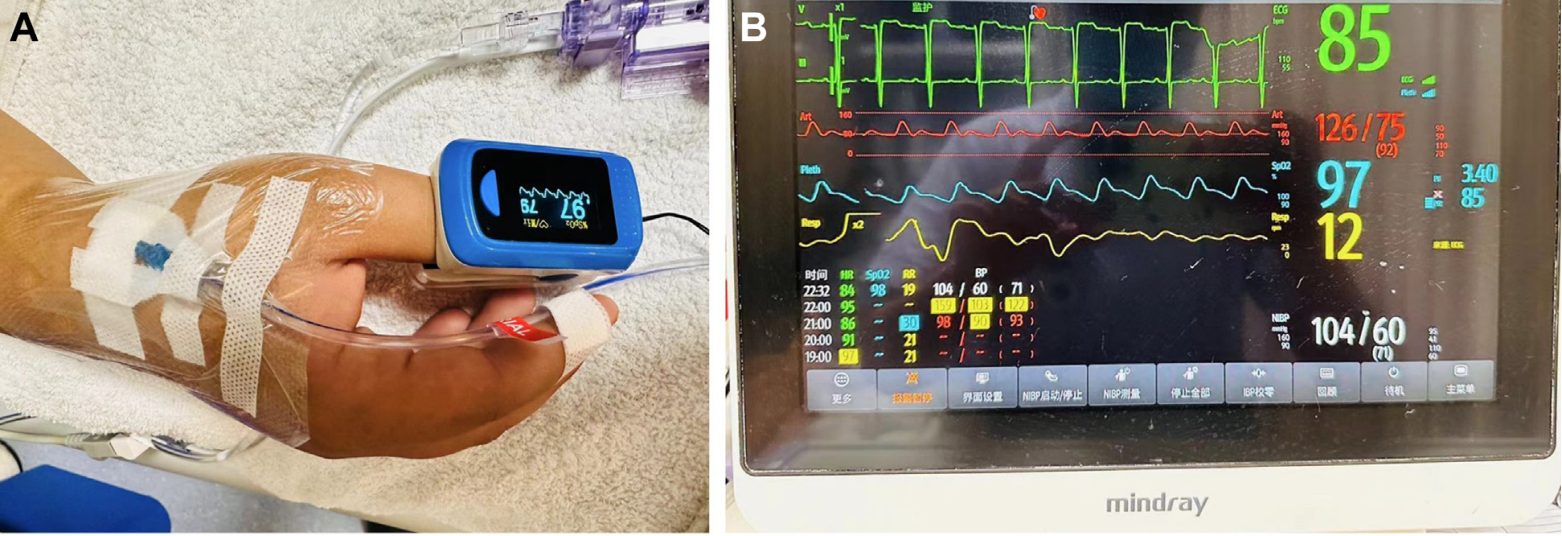
Invasive Arterial Blood Pressure Monitoring via Distal Radial Artery (Patient 3) (A) After cannulation of distal radial artery, patient’s blood pressure is stable as shown by thumb oxygen saturation monitoring. (B) Postprocedural vital signs monitoring shows consistent oxygen saturation levels between cannulated hand and noncannulated hand, confirming adequate perfusion and oxygenation.

The dRA puncture was performed similarly, with the catheter remaining in place for 5 days (Figure 3).

## Outcome and Follow-Up

### Patient 1

The catheter was removed after 3 days, when there were no signs of infection or radial artery occlusion. The patient was successfully weaned off vasopressors.

### Patient 2

The catheter was removed after 7 days with no complications. Follow-up observation indicated no arterial occlusion.

### Patient 3

The catheter was removed after 5 days without complications, and follow-up observation confirmed preserved wrist mobility and radial artery function.

In all 3 patients, hemostasis was achieved by applying a pressure bandage for 1 hour after the removal of the arterial catheter.

## Discussion

Recent studies support the use of dRA for arterial access, demonstrating success rates comparable with those conventional radial artery approaches, but with additional benefits such as reduced complication rates, extended catheter dwell times, and preservation of proximal radial artery function.^1^, ^2^, ^3^ In this report, we present 3 patients with acute coronary syndrome who successfully underwent invasive ABP monitoring via the distal radial artery, with both safety and effectiveness meeting clinical requirements. Given that the dRA approach requires mastering the puncture technique, we have provided a comprehensive demonstration of this method through illustrations and videos for reference by cardiologists, intensivists, and anesthesiologists. Our team has proficiently used this approach for routine coronary interventions,^4^ having completed >3,000 procedures, and we plan to conduct larger-scale clinical studies on ABP monitoring via dRA in the future.

The choice of the dRA as the primary puncture site is a well-considered decision. Although other access sites, such as the left or right radial artery, brachial artery, or femoral artery, can also be used for monitoring, each option comes with its unique risks. For instance, femoral artery puncture may lead to retroperitoneal hematoma, especially in patients receiving anticoagulation therapy. By contrast, dRA provides a stable puncture site that effectively reduces complications and allows for good hemostasis upon catheter removal. Another significant advantage of using the dRA method is the increased comfort and functionality for patients in the intensive care unit. Given that the puncture site is located in the anatomical snuffbox, patients can maintain full hand mobility, which is particularly important for nonsedated individuals. Patients can engage in daily activities such as using their phones or eating, thereby enhancing their overall experience in a critical care setting.

Regarding ultrasound guidance, we did not use this method in these cases, primarily because of the limitations in our operational equipment. To avoid the complications associated with puncture, we take a more cautious approach, which includes accurately assessing the arterial pulse location, using a modified Seldinger technique, avoiding forceful maneuvers, and applying adequate pressure after catheter removal. Although the current success rate for punctures is satisfactory, we acknowledge that incorporating ultrasound guidance in critically ill patients could further improve procedural success and reduce complications.

Concerning the risk of catheter kinking, whereas there may be concerns about this at the distal radial artery site, our experience thus far suggests that appropriate techniques can effectively mitigate this risk. By using the original needle as a guide and directly advancing the catheter, we significantly reduce the likelihood of kinking. We will continue to collect data in the future to further refine our technique, especially for patients with anatomical variations.

## Conclusions

Distal radial artery puncture is a safe and effective alternative for invasive ABP monitoring, particularly when preserving proximal radial artery function is crucial. Future research should explore the benefits of ultrasound guidance and further assess the applicability of dRA in hemodynamically unstable patients.

## Funding Support and Author Disclosures

This study is supported by the Capital's Funds for Health Improvement and Research (CFH 2024-2-4035), and Chinese Nursing Association Research Project (ZHKY202407). The authors have reported that they have no relationships relevant to the contents of this paper to disclose.

## Appendix

Video 1 The steps involved in performing a right distal radial artery puncture, including puncture site selection, local anesthesia, puncture technique, and catheter maintenance.
